# Rehabilitation outcomes of patients with posterior circulation stroke after acute inpatient rehabilitation

**DOI:** 10.3389/fresc.2026.1759128

**Published:** 2026-07-06

**Authors:** Dominic Chen, Shi Min Mah, Sheela James, Ee Ling Tay, Yee Sien Ng

**Affiliations:** 1 Department of General Medicine, Rehabilitation Medicine Service, Sengkang General Hospital, Singapore, Singapore; 2 Department of Physiotherapy, Sengkang General Hospital, Singapore, Singapore; 3 Department of Occupational Therapy, Sengkang General Hospital, Singapore, Singapore; 4 Department of Rehabilitation Medicine, Singapore General Hospital, Singapore, Singapore

**Keywords:** ambulation, functional independence measure (FIM), posterior circulation stroke, return to work, stroke rehabilitation

## Abstract

**Objective:**

We aim to describe functional outcomes, ambulation status, discharge disposition, and early return-to-work (RTW) at 3 months among patients with posterior circulation stroke (PCS) admitted to our acute inpatient rehabilitation unit.

**Design:**

This study employed a retrospective review of the medical records of patients.

**Setting and participants:**

The study included patients with posterior circulation stroke who were discharged from the acute inpatient rehabilitation unit between 1 August 2018 and 1 April 2020, with a total of 72 patients identified.

**Interventions:**

Patients received inpatient rehabilitation in the acute inpatient rehabilitation unit.

**Main outcome measures:**

Data collected included patients’ biodata, impairments, length of stay (LOS) in the acute ward and acute inpatient rehabilitation ward, functional independence measure scores on admission and on discharge to acute inpatient rehabilitation ward, rehabilitation disposition, and return to employment.

**Methods:**

We conducted a retrospective cohort study of consecutive adults with CT/MRI-confirmed posterior circulation stroke who were discharged from the inpatient rehabilitation unit between 1 August 2018 and 1 April 2020. The hospital had onsite neurosurgery provision but no in-hospital endovascular thrombolysis service during the study period. Multidisciplinary rehabilitation included roughly 1 h/day of physiotherapy and 1 h/day of occupational therapy, with speech therapy when indicated and daily reviews by the rehabilitation medicine specialist. The main outcomes were LOS in both the acute and rehabilitation wards, functional independence measure (FIM) scores at admission and discharge, ambulation status at discharge and at 3 months, discharge disposition, and RTW at 3 months. Descriptive statistics were used and logistic regression analysis was performed for RTW, using IBM SPSS Statistics 25.

**Results:**

Seventy-two patients were included in the study. The LOS in the acute medical ward was 9.7 days, while the LOS in the rehabilitation ward was 17.8 days. The mean total FIM gain was 12.4, while FIM efficiency was 1.01/day. At discharge, 77.8% of patients returned home. By 3 months, 90.3% of patients were living at home, while 5.6% were in nursing homes. By 3 months, 59.7% achieved independent ambulation (with or without a walking aid), 36.1% walked with assistance, and 4.2% were non-ambulant. RTW at 3 months was observed in 11.1% of the cohort. Younger age (odds ratio (OR) 0.75; 95% CI 0.576–0.975; *p* = 0.031; lower odds of RTW with increasing age) and higher discharge total FIM scores (OR 1.20; 95% CI 1.02–1.40; *p* = 0.026) were associated with RTW.

**Conclusions:**

In this retrospective study, structured inpatient rehabilitation was associated with functional gains in patients with posterior circulation stroke, and most patients were discharged home. Almost 60% achieved independent ambulation by 3 months. However, early RTW at 3 months was uncommon. These findings underscore the value of inpatient rehabilitation for PCS patients and highlight the ongoing need for gait or balance training and vocational rehabilitation pathways.

## Introduction

Posterior circulation stroke (PCS)—defined as an ischemic or hemorrhagic stroke affecting the brainstem, cerebellum, or occipital lobes—accounts for 20%–25% of all ischemic strokes (1). The prevalence of posterior circulation hemorrhagic stroke, however, is unknown. PCS results in sequelae such as weakness, sensory impairments, ataxia, dysarthria, vertigo, and cognitive impairment (2); these clinical presentations differ from strokes in other vascular territories, which may result in other neurological deficits such as aphasia, neglect, or cognitive impairment. Many patients who suffer posterior circulation strokes continue to suffer from these impairments, and rehabilitation remains an important approach to minimize the impact of these impairments. In a review of posterior circulation ischemic stroke, Dr. Alexander Ng highlighted that this area is relatively under-researched. While aspirin use and intravascular thrombolysis improved clinical outcomes, evidence remains inconclusive for intracranial vascular stenting and angioplasty (3). In a recently published clinical trial, the authors demonstrated that initial intravascular thrombolysis administration improved functional independence outcomes, measured using the modified Rankin Scale, compared with patients who did not receive thrombolysis. However, this study did not report whether the patients received intensive rehabilitation after the initial thrombolysis treatment, nor did it assess outcome measures like ambulation or return-to-work (RTW) status (4). Gait assessments at have also been used as a clinical screening tool to improve the triage process for patients who present with acute vertigo (5). Despite growing literature on acute management of posterior circulation ischemic stroke, including thrombolysis and endovascular therapy, there remains a paucity of data on inpatient rehabilitation outcomes specifically for PCS. Most stroke rehabilitation studies combine anterior and posterior circulation strokes or focus on global functional scales without detailing balance, gait, and vocational outcomes. To our knowledge, no previous study has reported functional independence measure (FIM) trajectories, ambulation status, and RTW specifically in PCS patients undergoing structured impatient rehabilitation. This limits the ability of rehabilitation teams to plan targeted interventions and counsel patients and families regarding expected recovery.

Therefore, the present study aims to describe the rehabilitation outcomes of ischemic and hemorrhagic posterior circulation stroke patients admitted to an acute inpatient rehabilitation unit in a regional hospital without in-house thrombolysis or endovascular treatment services. In particular, we sought to characterize functional gains (FIM), ambulation status at discharge and 3 months, discharge disposition, and early RTW at 3 months. By relating these outcomes to admission impairments, we aimed to provide clinicians with clinically relevant information to guide treatment planning, early goal-setting and prognosis discussion with patients and families.

## Methods

### Participants and eligibility

We retrospectively identified all consecutive adults (≥18 years) with CT- or MRI-confirmed ischemic or hemorrhagic PCS who were admitted to and discharged from the acute inpatient rehabilitation unit of our general hospital between 1 August 2018 and 1 April 2020. PCS was defined as a stroke occurring in territories supplied by the vertebral, posterior inferior cerebellar, basilar, or posterior cerebral arteries. Patients with strokes confined to anterior circulation territories were excluded. We did not restrict inclusion to first-ever strokes; patients with prior stroke were eligible if they were functionally independent or minimally dependent before the index event and met the admission criteria for inpatient rehabilitation. Admission criteria included medical stability, potential to benefit from intensive rehabilitation, and the ability to participate in at least 1 h/day of physiotherapy and occupational therapy. Patients with terminal illness or severe comorbidities that precluded active rehabilitation were not admitted to the unit and thus not included in the study.

### Assessments and outcome measures

#### Neurological and impairment assessments

Neurological deficits were extracted from routine clinical documentation at rehabilitation admission. Weakness was defined as a Medical Research Council (MRC) muscle strength grade ≤4 in at least one limb. Sensory impairment was defined as reduced pinprick, proprioception, or vibration in any limb compared with the contralateral side. Balance impairment was assessed using the Berg Balance Scale, with lower scores indicating poorer balance; scores below the age-adjusted normative range were coded as impaired. Visual field impairment (e.g., quadrantanopia or hemianopia) was diagnosed by the rehabilitation medicine team. Dysphagia and dysarthria were assessed by a speech therapist using standard bedside swallowing tests and speech evaluations. Behavioral changes (e.g., delirium, agitation, disinhibition, hallucinations) were documented by the multidisciplinary team.

#### Functional assessments

Functional status was measured using the FIM, which comprises 18 items across motor (13 items) and cognitive (5 items) domains. Each item is scored from 1 (total assistance) to 7 (complete independence), yielding total scores from 18 to 126. FIM was routinely recorded by trained rehabilitation staff at admission to and discharge from the rehabilitation unit. We calculated total FIM gain (discharge minus admission) and FIM efficiency (FIM gain divided by length of stay (LOS) in the rehabilitation unit in days).

#### Mobility and ambulation

Ambulation status was assessed at rehabilitation admission, discharge, and 3 months post-stroke. Independent ambulation without walking aid was defined as the ability to walk ≥50 m on level ground without physical assistance or a device. Independent ambulation with walking aid referred to walking ≥50 m with a single-point stick or other aid but without hands-on assistance. Ambulation with assistance was defined as the need for physical assistance from at least one person to walk up to 50 m, with or without a walking aid. Non-ambulant referred to patients who were unable to walk with or without assistance. These categories were documented by physiotherapists during routine assessments.

#### Return to work and follow-ups

Employment status prior to stroke and at 3 months post-stroke was extracted from medical and social work records. RTW was defined as resumption of part-time or full-time paid employment at 3 months, either in the same or a modified role. Follow-up at 3 months was determined from outpatient clinic visits and/or structured telephone calls when clinic data were unavailable. The 3-month time point was chosen *a priori* because the greatest gains in motor recovery and functional independence typically occur within this period.

### Rehabilitation interventions

All patients received a standardized multidisciplinary inpatient rehabilitation program. Physiotherapy was delivered for approximately 1 h per weekday and focused on lower-limb strengthening, balance and postural control, gait training (including over-ground walking, task-specific gait practice, and, when appropriate, stair training), and endurance exercises. Occupational therapy, also approximately 1 h per weekday, addressed upper-limb function, activities of daily living (ADL), transfers, and cognitive-perceptual strategies when needed. Speech therapy was provided for patients with dysphagia and/or dysarthria, targeting swallowing safety, dietary modification, and communication. Medical social workers assisted with discharge planning, caregiver training, and RTW counseling when relevant. All patients were reviewed daily by a rehabilitation medicine specialist-led team. Medical management followed prevailing stroke secondary prevention guidelines, including antithrombotic therapy, lipid-lowering agents, and blood pressure control as appropriate. Specific medication regimens were individualized but were not the focus of this analysis.

Descriptive statistics were analyzed using IBM SPSS Statistics 25. A bivariate logistic regression was performed to identify factors associated with return to work post-stroke at 3 months. Reporting adhered to STROBE guidelines for observational studies. This study was approved by the Singhealth Centralized Institutional Review Board (IRB Number 2020/2442).

## Results

A total of 72 patients were included in this study, with a mean age of 66 years. Of these, 59.7% were men and 40.3% were women. Before their stroke, 43.1% were employed. A history of stroke was present in 25% of the cohort. Vascular risk factors prior to stroke were common: 63.9% had a history of hypertension, 38.9% had diabetes mellitus, and 13.9% had atrial fibrillation. With regard to antithrombotic therapy, 26.4% were taking antiplatelet agents or anticoagulation prior to the index event. The majority of cases were ischemic strokes (80.6%, 58 patients), while 19.4% (14 patients) were hemorrhagic strokes. During the acute admission, clinical progression of stroke occurred in 8.3%, and 9.7% required neurosurgical intervention.

In terms of post-stroke impairments, the most common impairments were dysphagia (68.1%), weakness (69.4%) (defined as MRC grade 4 and below in the upper or lower limb), and motor coordination impairment (52.8%) (Table 1).

**Table 1:** Post-stroke impairments.

Impairment	Number of patients	Percentage
Visual impairment	4	5.6
Weakness	50	69.4
Sensory impairment	20	27.8
Motor coordination Impairment	38	52.8
Behavioral changes	6	8.3
Dysphagia	49	68.1
Dysarthria	36	50

The mean length of stay in the acute general ward was 9.72 days, while the mean LOS in the acute inpatient rehabilitation ward was 17.79 days. (Table 2) The wide range in acute ward LOS (2–38 days) reflected variations in stroke severity, acute medical complications, and logistical factors such as availability of rehabilitation beds. In terms of disposition post-acute inpatient rehabilitation, 19.4% of patients were transferred to a community hospital for further rehabilitation, 77.8% were discharged home, and two patients were discharged to nursing homes. At 3 months post-stroke, 2.8% of patients remained in a community hospital, which is a tier 2D rehabilitation facility under the National ONE Rehab framework (13), 1.4% had died, 90.3% went home, and 5.6% were in nursing homes (Table 2).

**Table 2:** Length of stay and FIM scores.

Length of stay and FIM scores	Mean (range; SD)
Length of stay in acute ward in days	9.72 (2–38; 6.358)
Length of stay in acute inpatient rehabilitation in days	17.79 (2–135; 20.726)
Admission motor FIM	39.49 (11–72; 14.125)
Admission cognitive FIM	28.00 (5–35; 8.395)
Admission total FIM	73.76 (18–119; 23.574)
Discharge motor FIM	48.75 (11–72; 14.783)
Discharge cognitive FIM	29.35 (7–35; 7.147)
Discharge total FIM	86.12 (25–120; 22.913)
Discharge FIM − admission FIM	12.36 (−7–46; 11.965)
FIM gain/rehab length of stay in days	1.0092 (−1.75–11.67; 1.55387)

The mean admission total FIM score was 73.76, while the mean total discharge FIM score was 86.12. FIM efficiency (defined as FIM gain divided by rehabilitation ward length of stay in days) was 1.01.

Most patients (90.3%) were home 3 months post-stroke, with the rest of the dispositions as of 3 months displayed in Table 3.

**Table 3:** Discharge disposition post-rehabilitation [disposition at 3 months post-stroke (death/home/community hospital/nursing home)].

Location	Number of patients	Percentage
Community hospital	2	2.8
Death	1	1.4
Home	65	90.3
Nursing home	4	5.6

In terms of ambulation status after admission to inpatient rehabilitation, only one person walked independently, while the rest ambulated with assistance or were non-ambulant. By 3 months, 59.7% of patients walked independently with or without a walking aid, while 36.1% walked with assistance (Table 4a). Independent ambulation was defined as the ability to walk 50 m without a walking aid and without any assistance from a person. Ambulation with assistance was defined as walking up to 50 m with the help of one person, with or without a walking aid.

**Table 4:** Ability to ambulate.

(a) Ability to ambulate at admission		
Ambulation status	Number of patients	Percentage
Ambulate independently without walking aid	1	1.4
Ambulate with assistance	45	62.5
Not ambulating	26	36.1

(b) Ability to ambulate at 3 months		
Ambulation status	Number of patients	Percentage
Ambulate independently with walking aid	17	23.6
Ambulate independently without walking aid	26	36.1
Ambulate with assistance	26	36.1
Not ambulating	3	4.2

(c) Ability to stand unsupported and ability to ambulate at 3 months			
Ambulation status	Able to stand unsupported: No	Able to stand unsupported: Yes	Total
Ambulate independently with walking aid	12	5	17
Ambulate independently without walking aid	15	11	26
Ambulate with assistance	22	4	26
Not ambulating	3	0	3
Total	52	20	72

(d) Ability to sit unsupported on admission to inpatient rehabilitation and ability to ambulate at 3 months			
Ambulation status	Able to sit: no	Able to sit: yes	Total
Ambulate independently with walking aid	5	12	17
Ambulate independently without walking aid	0	26	26
Ambulate with assistance	11	15	26
Not ambulating	3	0	3
Total	19	53	72

(e) Presence of weakness on admission to inpatient rehabilitation and ability to ambulate at 3 months			
Ambulation status	Weakness: no	Weakness: yes	Total
Ambulate independently with walking aid	5	12	17
Ambulate independently without walking aid	12	14	26
Ambulate with assistance	5	21	26
Not ambulating	0	3	3
Total	22	50	72

Tables 4c–e present cross-tabulations of ambulation outcomes against the presence of weakness, ability to sit unsupported, and ability to stand unsupported. Weakness was present in 69.4% (50/72) of patients, inability to sit unsupported was observed in 26.4% (19/72), and inability to stand unsupported was documented in 74.3% (52/70). At 3 months post-stroke, 59.7% (43/72) of patients achieved independent ambulation with or without a walking aid. Of the 53 patients able to sit unsupported, 38 (71.69%) were able to walk without assistance at 3 months, with or without a walking aid. Of the 20 patients able to stand unsupported, 16 (80%) were able to ambulate without assistance at 3 months, with or without a walking aid. Of the 22 patients who did not have weakness, 17 (77%) were able to ambulate without assistance at 3 months, with or without a walking aid. Out of the 50 patients with weakness, 26 (52%) patients were able to ambulate without assistance at 3 months, with or without a walking aid.

On the employment front, 43.1% of patients were employed prior to the stroke. At 3 months post-stroke, only 11.1% of patients returned to employment. Using returning to work by 3 months as an outcome variable, binary logistic regression analysis was performed using IBM SPSS Version 25 for the variables listed in Table 5b as these were factors that have been studied in recent paper regarding return to work after stroke (19). It was found that younger age (odds ratio (OR) 0.75, 95% CI 0.576–0.975, *p* = 0.031) and higher total discharge FIM score (OR 1.198, 95% CI 1.022–1.404, *p* = 0.026) were independently associated with patients returning to work.

**Table 5:** Return to employment 3 months post-stroke.

(a) Return to employment 3 months post-stroke		
Return to employment at 3 months	Frequency	Percent
No	64	88.9
Yes	8	11.1
Total	72	100.0

(b) Odds ratio of factors related to return to work			
Factor	Odds ratio	*P* value	95% CI
Younger age	0.75	0.031	0.576–0.975
Employment prior to stroke	0.34	0.065	0.001–1.229
Total discharge FIM	1.198	0.026	1.022–1.404
Admission total FIM	0.886	0.145	0.753–1.043
Ability to ambulate at admission	0.314	0.609	0.004–26.737
Length of stay in acute ward in days	0.775	0.325	0.466–1.287
Length of stay in acute inpatient rehabilitation in days	0.7	0.047	0.492–0.996

## Discussion

In this study, approximately 60% of patients were men, with the rest being women, consistent with the slightly higher incidence of PCS in men than women. The proportion of ischemic posterior circulation stroke patients was higher than the 63% quoted in another study (6). The rate of progression of stroke in our study was 8.3%, which is lower than the 16%–33% reported in another study examining patients with ischemic posterior circulation stroke with recurrence of stroke within 90 days of the first stroke (7). A high index of suspicion is therefore needed for recurrence or progression of stroke when managing such patients in the acute inpatient rehabilitation period.

The three most common impairments post-stroke in our cohort were weakness, dysphagia, and motor coordination impairment, findings that align with the New England Medical Center Posterior Circulation Registry (8).

The mean length of acute ward stay was 9.72 days and inpatient rehabilitation ward length of stay was 17.79 days, similar to the length of stay of acute stroke patients in similar settings in a local study (9).

FIM efficiency gain was approximately 1 point a day during inpatient rehabilitation, demonstrating the value of inpatient-based rehabilitation for posterior circulation stroke in Singapore. This is better than the average 0.48 FIM efficiency gain described by Woo et al. in a 2008 analysis of inpatient stroke rehabilitation efficiency (10). Greater FIM efficiency gain during inpatient stroke rehabilitation has also been associated with higher rates of home discharge (11). This appears to be corroborated by the relatively high rate of patients discharged home in this study, at 77.8%. Notably, approximately 19.4% of patients still needed to go to the community hospital for further rehabilitation, which is fairly similar to the rates of discharge patterns reported in another study (12). This strengthens the argument for a neuro rehabilitation cohort in community hospitals, in line with the Singapore Ministry of Health's National One Rehabilitation Framework (13).

With inpatient rehabilitation, the proportion of patients ambulating without assistance rose from 1.4% on admission to the rehabilitation unit to 59.7% on discharge from inpatient rehabilitation. However, 40.3% of patients could not walk without assistance on discharge from inpatient rehabilitation. Possible reasons include greater baseline stroke severity in patients referred for inpatient rehabilitation, the high prevalence of dysphagia and coordination deficits, and involvement of brainstem and cerebellar structures critical for postural control and locomotion in PCS. Another study on acute ischemic stroke patients noted that the ability of patients to sit unsupported by 72 h strongly correlated with the ability to walk independently by 6 months post-stroke (14). In our study, many patients who were unable to stand unsupported at admission to the rehabilitation ward were able to ambulate after structured inpatient rehabilitation, although most required the use of a walking aid. In another study looking at acute stroke patients in all vascular territories done in 5 hospitals in Japan, it was found that the probability of walking independence for patients admitted to inpatient rehabilitation with Brunstromm recovery scale for lower extremity (BRS-LE) (charted on admission to rehabilitation) of 4-6 was 66.7% and Ability of Basic Movement Scale 2 (ABMS-T) of 4-6 was 73.8%, which is higher than our study, possibly because this study excluded all patients who were initially more disabled with a modified Rankin Scale of 3 and above (15). For posterior circulation stroke patients with weakness in our cohort, slightly more than half were able to ambulate independently at 3 months, with or without a walking aid. Other studies have found that admission motor and balance assessments are significant predictors of independent walking (16). Future studies can also explore the role of biomarkers, including computed tomography-derived prediction scores (17) and transcranial magnetic stimulation (18). Community-based ambulation strategies can be considered after inpatient rehabilitation (19).

The rate of patients returning to work in this study was 11.1% at 3 months. Most of these patients worked prior to the stroke. Other local studies have reported return-to-work post-stroke (for strokes in all vascular territories) rates between 39% and 55%, although this may have been measured beyond 3 months (20). In our study, patients of a younger age and with a higher total discharge FIM score were more likely to return to work at 3 months. This contrasts with findings from another retrospective study, which identified premorbid office employment, discharge ambulation FIM >5, discharge cognitive FIM >25, and lower Charlson Comorbidity Index scores at discharge as positive predictors of successful return to work (21). Ibikunle et al. described the validation of a return-to-work assessment tool for post-stroke patients (22), which could be applied in future studies investigating return to work following posterior circulation stroke. Teo et al. advocated for early identification of factors that may influence long-term RTW outcomes, so that efforts can be more targeted, thereby increasing the chance of post-stroke RTW (23).

## Study limitations

This study has several limitations. The retrospective design is subject to measurement bias and unmeasured confounding. Selection bias is likely, because more severe posterior circulation stroke patients tend to be admitted to inpatient rehabilitation. The small number of RTW events increases the risk of overfitting in regression models and contributes to wide confidence intervals. Finally, these results reflect a single-center experience without in-house endovascular services during the study period, which may limit generalizability.

## Conclusion

We report functional, ambulation, and RTW outcomes for PCS patients undergoing structured inpatient rehabilitation in a single regional hospital. Most patients achieved meaningful FIM gains and were living at home by 3 months, and approximately 60% walked independently, with or without a walking aid, highlighting the value of multidisciplinary inpatient rehabilitation for this population. However, a substantial minority remained dependent for walking, and early RTW was uncommon, emphasizing the need for ongoing outpatient and community-based gait and balance training, as well as the development of dedicated vocational rehabilitation pathways. Larger, multicenter studies are needed to confirm these findings, refine prognostic markers for walking and RTW, and identify the most effective rehabilitation strategies for PCS patients.

## Data Availability

The raw data supporting the conclusions of this article will be made available by the authors without undue reservation.
